# Joint Effects of Physical Activity and Body Mass Index on Prevalent Diabetes in a Nationally Representative Sample of 1.9 Million US Adults

**DOI:** 10.1155/jdr/7466757

**Published:** 2025-03-08

**Authors:** David Abernethy, Jason Bennie, Toby Pavey

**Affiliations:** ^1^School of Exercise and Nutrition Science, Queensland University of Technology, Brisbane, Queensland, Australia; ^2^Murrumbidgee Primary Health Network, Wagga Wagga, New South Wales, Australia; ^3^Centre for Primary Health Care and Equity, Faculty of Medicine and Health, University of New South Wales, Sydney, Australia; ^4^Faculty of Science and Health, School of Rural Medicine, Charles Sturt University, Dubbo, Australia; ^5^Faculty of Health, Queensland University of Technology, Brisbane, Queensland, Australia

## Abstract

**Aim:** To investigate the joint effects of physical activity (PA) and body mass index (BMI) on prevalent diabetes mellitus in a nationally representative sample of US adults.

**Materials and Methods:** Data were pooled from five US Behavioral Risk Factor Surveillance System (BRFSS) surveys from 2011 to 2019. Cross-sectional associations between independent and combined PA and BMI status and diabetes were analysed using Poisson's log-linear regression with a robust-error variance, reported by adjusted prevalence ratios (APRs). These models were adjusted for relevant sociodemographic, behavioral, and health-related factors.

**Results:** Data was available for 1,913,732 individuals (≥ 18 years). Considering individuals highly active and with normal weight as the reference group, there was an association between decreasing levels of PA and increasing BMI and diabetes prevalence. APRs ranged from APR = 1.09 (nonactive, normal weight group; 95% CI = 1.09–1.09), 1.67 (nonactive, overweight group; 95% CI = 1.67–1.67), 2.23 (nonactive, Class I obesity group; 95% CI = 2.23–2.23), 2.71 (nonactive, Class II obesity group; 95% CI = 2.71–2.71), and 3.17 (nonactive, Class III obesity group; 95% CI = 3.16–3.17).

**Conclusions:** BMI appears to be a substantially larger predictor of diabetes compared to PA in a large population-level sample of US adults. PA provided modest reductions in the prevalence of diabetes but did not attenuate the detrimental impact of overweight and increasing levels of obesity on diabetes prevalence.

## 1. Introduction

Obesity has been well-established as a risk factor for chronic disease and other adverse health complications [1]. This association is deeply intertwined with Type 2 diabetes (T2D) mellitus (T2DM), a chronic health condition typified by insulin resistance, beta-cell dysfunction, and elevated blood glucose that causes gradual harm to the cardiovascular, kidney, nervous, and ocular systems [2, 3]. T2D accounts for approximately 90% of all diagnosed cases of diabetes mellitus [4]. In contrast, Type 1 diabetes (T1D) mellitus (T1DM) makes up about 5%–10% of these cases [5], with the remaining cases attributed to other less common types of diabetes mellitus.

In 2021, 52.2% of all global T2D disability-adjusted life years (DALYs) were attributable to elevated body mass index (BMI) alone, and the contribution of high BMI to T2D DALYs increased by 24.3% worldwide between 1990 and 2021 [6]. The disproportionate risk of developing T2D across various levels of adiposity was highlighted in a systematic review and meta-analysis of 26 million adults from 216 cohort studies. In that study, it was found that increases in BMI and waist circumference were associated with substantially elevated relative risks (RRs) of T2D (RR > 1.6), indicating the strong positive association between BMI, central obesity measures, and T2D risk [7].

In 2019, there were 437.9 million prevalent cases of T2D, 1.5 million deaths, and 66.3 million DALYs due to this condition [8], causing the disease to rank ninth among the leading causes of mortality [9]. Based on International Diabetes Federation (IDF) estimates, the global prevalence of diabetes in 20–79-year-olds in 2021 was about 10.5% (536 million people) and is expected to rise to 12.2% (783.2 million) in 2045 [10]. The economic burden of diabetes is also considerable, since global health expenditure related to diabetes was approximately $966 billion for adults aged 20–79 years in 2021 and is expected to surpass $1 trillion by 2045 [10]. In the United States alone, $412 billion is spent each year diagnosing and managing patients with diabetes, with medical expenditure approximately 2.6 times higher for these individuals compared to those without diabetes [11].

Physical activity (PA) plays a pivotal role in combating the increasing prevalence of obesity and consequently reducing the incidence of T2D, establishing itself as a notable prevention tool due to its cost-effective and noninvasive nature. Epidemiological evidence has emphasized the protective benefits of PA against chronic disease [1, 12] and all-cause and disease-specific mortality [13, 14], in a dose-response manner. For T2D, risk reductions have been established in systematic reviews and meta-analyses examining the protective benefits of PA. Smith et al. [15] discovered that adherence to current public health recommendations of 150 min of moderate PA a week yielded a risk reduction of 26% of T2D compared with being inactive. The global age-standardised prevalence of individuals who are insufficiently active according to the World Health Organisation's PA guidelines amounted to 27.5% in 2016, with this percentage varying across countries and geographical regions [16]. Within the United States, the prevalence of insufficient PA is even greater, with estimates ranging from 49.5% to 65.2% [17, 18].

While the individual effects of physical inactivity and BMI as risk factors for T2D are well-recognized and have been heavily investigated, research efforts into their joint effects are relatively limited. A meta-analysis of nine prospective cohort studies best reflects the current foundation of evidence, indicating that people with overweight or obesity with low PA were at an increased risk of developing T2D [19]. However, a limitation with that study was similar to many others investigating the joint effects of PA and BMI on diabetes, as obesity was not separated into three individual classes and limited confounding variables were incorporated (age, gender, level of education, and smoking status). As a consequence, existing research has not entirely encapsulated the association between PA and BMI on diabetes across all levels of obesity while also accounting for key confounding factors. Our previously published abstract [20] analysing the same population used binary logistic regression, whereas this study employs Poisson's log-linear regression with a robust error variance—a more appropriate and interpretable measure for cross-sectional data. Therefore, this study is aimed at examining the joint effects of PA and BMI on prevalent diabetes in a nationally representative sample of 1.9 million US adults while incorporating all classes of obesity and relevant sociodemographic, behavioral, and health-related confounding variables.

## 2. Methods

A cross-sectional survey developed in 1984, the Behavioral Risk Factor Surveillance System (BRFSS), is conducted each year in the United States [21]. It is the world's largest continuously conducted health survey and collects data from residents regarding health-related risk behaviors, preventive health services, and chronic health conditions [21]. In the current study, data from the 2011, 2013, 2015, 2017, and 2019 BRFSS surveys were pooled together for analysis. Each survey is approved by the National Center for Health Statistics Research Ethics Review Board [21]. The BRFSS did not require ethical approval for use, as per BRFSS guidelines [22]. For the 2011, 2013, 2015, 2017, and 2019 BRFSS surveys, the response rates were 49.7%, 45.9%, 47.2%, 45.9%, and 49.4%, respectively [21].

Across each BRFSS survey, the same survey and methodology were used to assess self-reported PA, anthropometric measures, and sociodemographic and behavioral variables [21]. The large scope and depth of questioning of the BRFSS have meant its surveys are often utilised in population health research to explore relationships between variables and their associations with health outcomes [21]. Numerous explorations and applications of the BRFSS have noted that all survey questions are either moderately or highly reliable and valid, while having data comparable to similar population health surveys [23, 24]. Each respondent was provided with a unique weighting factor, which helps correct for clustering, stratification, and nonresponse in analyses, increasing sample representativeness and reducing the risk of bias. Details of the weighting process for respondents are outlined elsewhere [25].

Data was available for 2,307,980 respondents; however, for the current analyses, participants were excluded if they were missing data for PA (*n* = 194,089), BMI (*n* = 101,793), or both variables (*n* = 61,603). Individuals classified as “underweight” (BMI < 18.5 kg/m^2^) were also excluded (*n* = 36,763) to avoid considering subjects with poor health related to weight loss and undernutrition [26]. To enhance the generalizability of this research, no other inclusion or exclusion criteria were applied to the initial sample. The final sample available for analysis included 1,913,732 respondents.

### 2.1. PA Assessments

Moderate to vigorous physical activity (MVPA) of BRFSS respondents was assessed using previously validated questionnaires [27]. Cohen's kappa coefficient (Cohen's *k*) is a statistical measure used to assess the level of agreement between two raters or evaluators on categorical data, while accounting for agreement that could occur by chance [28]. The survey questions have acceptable test–retest reliability (Cohen's *k* = 0.67–0.84) and concurrent validity (Cohen's *k* = 0.17–0.22) for classifying groups to levels of recommended MVPA when compared to accelerometry as the gold standard [27].

To examine PA habits of individuals, the BRFSS surveyors presented respondents with the declaration: “The next few questions are about exercise, recreation or physical activities other than your regular job duties.” They followed by asking “During the past month, other than your regular job, did you participate in any physical activities or exercises such as running, callisthenics, golf, gardening or walking for exercise?”. If respondents indicated they participated in PA, the surveyors followed up with questions about the type, frequency, and duration of this activity: “What type of physical activity or exercise did you spend the most time doing during the past month?”, “How many times per week or per month did you take part in this activity during the past month?”, and “When you took part in this activity, for how many minutes or hours did you usually keep at it?”

The same questions were given to respondents about a second activity when they indicated engaging in more than one type of PA. All time reported being physically active was reported in hours and minutes, with each activity coded as “aerobic” or “nonaerobic” according to a list of 56 activities [29, 30]. Aerobic activities included exercises such as walking, running, cycling, and swimming, whereas nonaerobic activities included those such as bowling, weight training, house chores, and golf. All aerobic activities were included as MVPA and classified as moderate or vigorous according to the estimated metabolic equivalent of task (MET) [21]. MET is a way to measure the energy required during PA, with activities assigned MET values based on how much energy they demand compared to resting (1 MET), with higher values indicating more intense activities [31]. Moderate-intensity activities were defined by a MET value ranging between 3.0 and 5.9, whereas vigorous-intensity activities were defined by a MET value ≥ 6.0 [32]. Total MVPA was calculated by multiplying weekly minutes of vigorous-intensity activity by two and then adding this to weekly minutes of moderate-intensity activity, in accordance with established guidelines [33, 34].

### 2.2. PA Categories

Four mutually exclusive PA categories were formed according to participant adherence to the World Health Organization's aerobic MVPA guidelines: (i) “nonactive” (MVPA = 0.00 min/wk); (ii) “inactive” (MVPA = 0.01 − 149.99 min/wk); (iii) “active” (MVPA = 150.00 − 299.99 min/wk); or (iv) “highly active” (MVPA ≥ 300.00 min/wk) [35]. These guidelines were developed by synthesising high-quality evidence from longitudinal cohort studies that have been shown to reduce the risk of adverse health outcomes and increase life expectancy [35, 36].

### 2.3. BMI Categories

BMI data was derived from self-reported height (meters) and weight (kilograms) by dividing the body weight in kilograms by the height in meters squared using the standardised formula: BMI = kg/m^2^. A previous study has shown a strong correlation (*r* = 0.95) between self-reported BMI and objectively measured BMI [37]. Five standard, mutually exclusive BMI categories were used: (i) “normal weight”: 18.5–24.9 kg/m^2^; (ii) “overweight”: 25.0–29.9 kg/m^2^; (iii) “Class I obesity': 30.0–34.9 kg/m^2^; (iv) “Class II obesity': 35.0–39.9 kg/m^2^; and (v) “Class III obesity': ≥ 40.0 kg/m^2^.

### 2.4. Combined PA and BMI Categories

According to an individual's PA and BMI classifications, 20 combined PA-BMI categories were created. These categories ranged from “normal weight and highly active” (considered as the reference group) to “Class III obesity and nonactive'. Table S1 outlines the categories to which individuals were allocated.

### 2.5. Outcome Variable

To assess an individual's diabetes status, study participants were provided with the statement “Have you ever been told you have diabetes?” [21]. Previous studies have included this condition as an outcome variable when examining the individual effects of PA and BMI on health [38, 39]. This survey question did not distinguish between T1D and T2D. However, women diagnosed with gestational diabetes were not included as prevalent cases due to their pregnancy. Previous research has indicated that self-reported cases of diabetes have shown substantial agreement with verified medical records [40].

### 2.6. Potential Confounders

Sociodemographic, lifestyle, and health characteristics were assessed using standardised survey items. Each of the confounding variables included has been recognized as having an association with BMI, PA, or diabetes [41, 42]. Sociodemographic variables included age (18–24, 25–34, 35–44, 45–54, 55–64, and 65 years or older), sex (male and female), level of education (did not graduate high school, graduated high school, attended technical school or college, and graduated technical school or college), race (White, Black, Multiracial, Hispanic, and Others), employment status (employed, unemployed, homemaker, student, retired, and unable to work), marital status (married/member of an unmarried couple, divorced/widowed/separated, and never married), and number of children (zero, one, two, three, or more). Lifestyle variables included smoking status (current smokers, former smokers, and persons who never smoked), muscle-strengthening exercise frequency (sessions per week), and fruit and vegetable consumption (number of daily servings). The BRFSS measures daily fruit and vegetable consumption with six items assessing the frequency of consuming 100% fruit juice, fruit, beans (legumes), dark green vegetables, orange vegetables, and other vegetables for the month before respondent interviews [21]. Health-related variables included self-rated health (excellent, very good, good, fair, and poor), number of poorer mental health days, and number of chronic health conditions. Days with poor mental health were assessed by asking respondents “Now thinking about your mental health, which includes stress, depression, and problems with emotions, for how many days during the past 30 days was your mental health not good?” (0, 1–2, 3–6, 7–14, and 15–30). The presence of 11 other chronic health conditions assessed includes hypertension, high blood cholesterol, heart attack, coronary heart disease, stroke, asthma, cancer (excluding skin cancer), chronic obstructive pulmonary disease, depression, kidney disease, and arthritis. These conditions were included due to their recognized association with morbidity and mortality [43, 44] and were assessed by asking respondents “Has a doctor, nurse or other health professionals ever told you that you had any of the following?” [21].

### 2.7. Statistical Analysis

All statistical analyses were completed using IBM SPSS Statistics software Version 29.0. In the analysis, to increase population representativeness, weighting factors were implemented to correct for nonresponse, stratification, and clustering [25]. Descriptive statistics for both the explanatory, outcome, and confounding variables were derived and reported by both the crude number of individuals in each category (*n*) and the weighted proportion (and 95% confidence intervals) of individuals relative to the total sample population.

The associations between joint PA-BMI subcategories and prevalent diabetes were investigated using Poisson's log-linear regression model with a robust error variance. Adjusted prevalence ratios (APRs) were calculated for each joint PA-BMI subcategory compared to the “normal weight and highly active” group (reference group). All models were adjusted for relevant confounding variables. Stratified analyses for both PA and BMI were also completed to examine the effects of each explanatory variable with a reference group in each subcategory (normal weight across PA thresholds and highly active across BMI categories). The use of prevalence ratios obtained from Poisson regression is considered a more statistically robust method in cross-sectional epidemiologic studies, compared to general logistic regression [45]. *p* values were based on two-sided tests and were considered statistically significant at *p* < 0.05. Before performing analyses, collinearity between the various explanatory variables was examined using variance inflation factor (VIF), with VIF values ≥ 2.5 indicating multicollinearity [46]. VIF values ranged from 1.05 to 2.14, displaying no evidence of collinearity.

## 3. Results

Data was available for 1,913,732 individuals (≥ 18 years old) as displayed in Table 1. In summary, 20.0% of respondents were aged 65 years or older, 49.4% were female, and the majority were identified as either White, Black, or Hispanic (92.4%). Over half of the sample had never smoked (57.7%), and 18.4% self-reported having “excellent” health. For PA, 28.5% of the sample were “nonactive,” 20.7% were “inactive,” 19.0% were “active,” and 31.8% were “highly active.” With regard to BMI categories, 33.8% of participants had normal weight, 36.2% were overweight, and 30.1% were people with different classes of obesity. A total of 10.7% (*n* = 252,198) of the sample reported they had been diagnosed with diabetes.

### 3.1. Individual Effects of PA and BMI on Prevalent Diabetes

The individual effects of PA and BMI on prevalent diabetes are reported in Table S2. The APRs for diabetes modestly changed according to the PA category, with statistically significant differences between categories evident. Compared to those who were highly active, the prevalence of diabetes was higher for active (APR = 1.06, 95% CI = 1.06–1.06), inactive (APR = 1.10, 95% CI = 1.10–1.10), and nonactive individuals (APR = 1.11, 95% CI = 1.10–1.11). The prevalence of diabetes was significantly (*p* < 0.01) higher among individuals classified as affected by overweight (APR = 1.54, 95% CI = 1.54–1.54), Class I obesity (APR = 2.12, 95% CI = 2.12–2.12), Class II obesity (APR = 2.66, 95% CI = 2.66–2.66), and Class III obesity (APR = 3.11, 95% CI = 3.11–3.12) as compared to that observed in normal weight individuals.

### 3.2. Joint Effects of PA and BMI on Prevalent Diabetes

**Figure 1:**
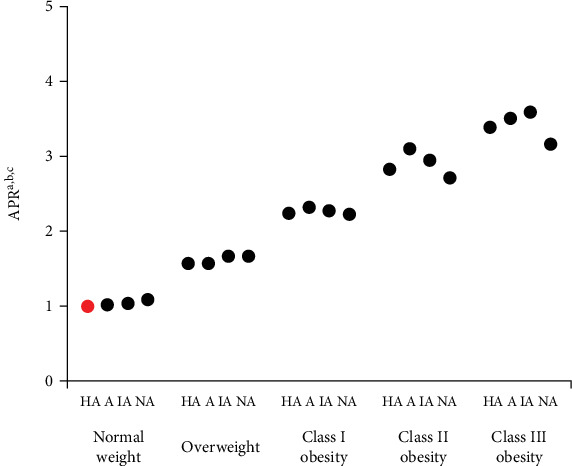
Adjusted prevalence ratios^a,b^ (APRs) for prevalent diabetes by PA-BMI categories^c^. Prevalent diabetes is defined as respondents having been told they have diabetes by a doctor, nurse, or health professional. The red point indicates the highly active, normal weight reference group, with an adjusted prevalence ratio of 1. The black points indicate the remaining 19 PA, body mass index categories outlined in Table S1 and their corresponding adjusted prevalence ratios. The study design of the Behavioral Risk Factor Surveillance System was accounted for by incorporating the sample weighting variable during analysis. ^a^95% confidence intervals are too precise to be seen on the graph. ^b^Adjusted for age, sex, race, education, employment status, marital status, number of children, smoking status, fruit and vegetable intake, self-rated health, days of poorer mental health, number of additional chronic health conditions, and muscle-strengthening exercise frequency. ^c^HA, highly active; A, active; IA, inactive; NA, nonactive.

The model examining the joint effects of PA and BMI on prevalent diabetes after adjusting for all confounding variables can be seen in Figure 1. Compared to the reference group (highly active and normal weight group), there was a linear trend relationship between decreased PA and increased BMI in prevalent diabetes. APRs ranged from APR = 1.09 (nonactive and normal weight group; 95% CI = 1.09–1.09), 1.67 (nonactive, overweight group; 95% CI = 1.67–1.67), 2.23 (nonactive, Class I obesity group; 95% CI = 2.23–2.23), 2.71 (nonactive, Class II obesity group; 95% CI = 2.71–2.71), and 3.17 (nonactive, Class III obesity group; 95% CI = 3.16–3.17). Among individuals living with Obesity Class II, those who were nonactive had a lower prevalence of diabetes (APR = 2.71, 95% CI = 2.71–2.72) compared to their active (APR = 3.10, 95% CI = 3.10–3.11) or inactive (APR = 2.96, 95% CI = 2.95–2.96) counterparts. Similarly, among those living with Obesity Class III, nonactive individuals (APR = 3.17, 95% CI = 3.16–3.17) had a lower prevalence of diabetes compared to those who were active (APR = 3.51, 95% CI = 3.51–3.52) or inactive (APR = 3.60, 95% CI = 3.59–3.60). These results were statistically significant (*p* < 0.01) for all joint PA and BMI categories aside from the categories “normal weight and physically active” and “normal weight and physically inactive.”

The first stratified analysis examined the potential protective effects of PA, with highly active individuals serving as the reference group across each BMI category (Table 2). In each of the BMI categories, the protective effects of being highly active were statistically significant (*p* < 0.01), as engaging in less than 300 min of MVPA a week was associated with a modestly increased prevalence of diabetes. Large increases in the prevalence of diabetes were observed among individuals in all obesity classes, particularly among active and inactive individuals (APR range = 1.03–1.09).

The second stratified analysis examined the effects of BMI, with individuals with normal weight serving as the reference group across each PA quartile (Table 3). In each of the PA quartiles, the impact of excess body weight was significantly noticeable (*p* < 0.01), as increasing BMI levels were associated with a substantially increased prevalence of diabetes. This was observed across each PA quartile, as individuals with Class I (APR range = 2.02–2.19), Class II (APR range = 2.47–2.87), or Class III (APR range = 2.88–3.32) obesity experienced significantly higher prevalence of diabetes compared to individuals with normal weight (Table 3).

## 4. Discussion

This study investigated the joint effects of PA and BMI on prevalent diabetes in a nationally representative sample of US adults. The key finding from this study was that a joint effect of physical inactivity and increased BMI on prevalent diabetes was observed among US adults, as the most physically active and lowest BMI individuals had the lowest prevalence of diabetes. Although PA was associated with reduced diabetes prevalence, it modestly attenuated the increased APR for diabetes attributable to excess BMI. To our knowledge, this is the first US nationally representative study to investigate the joint effects of PA and BMI on diabetes while exploring all obesity categories and adjusting for a range of sociodemographic, behavioral, and health variables.

A 2004 study by Weinstein et al. [47] was a foundational investigation into the relationship between PA, BMI, and T2D, inspiring subsequent research, including the current study. Consistent with findings reported by Weinstein et al. [47], this study found that BMI is the dominant factor for diabetes, with PA offering modest protective effects against the increased likelihood associated with higher BMI. Expanding on this foundational work and subsequent research, the current study utilised a nationally representative sample of 1.9 million US adults, enhancing generalisability and providing a more nuanced understanding of how PA influences diabetes prevalence among different BMI categories.

While it appears that PA has only a modest effect in reducing the prevalence of diabetes in this study, this effect was more noticeable among individuals with normal weight and overweight compared to those with Class I, Class II, or Class III obesity. A previous study by Warner et al. found that persons living with obesity misclassified PA intensity to a greater extent than individuals with normal weight and overweight, with a lack of agreement between objective and self-reported measures of PA when classifying individuals based on PA recommendations [48]. Accordingly, it is plausible that the lack of protective effect of PA against diabetes among individuals with Class I, Class II, or Class III obesity in this study was impacted by overestimations of PA levels that are more common among individuals living with obesity [48].

The results from this study are consistent with previous observational studies investigating the impact of the joint association between PA and obesity on the risk of diabetes (particularly T2D), indicating that excess body weight is a greater risk factor for T2D compared to physical inactivity [19, 49–53]. A study that also incorporated diet score when examining joint effects of PA and BMI has highlighted the substantial influence of BMI on T2D. The population attributable risk (PAR) for T2D associated with the combined effects of lower PA, higher BMI, and a less healthy diet was 52.7% and 58.4% for men and women, respectively. Notably, BMI appeared to be the most significant contributor, as the PARs for BMI and diet were similar to those for BMI and PA, while PA and diet together had a substantially lower PAR [54].

The elevated likelihood of diabetes attributable to increased BMI compared to varying levels of PA has also been observed in two systematic reviews and in a meta-analysis [19, 50, 51]. Studies on diabetes included in the review published by Fogelholm [50] found that individuals with obesity, even with a high PA level, were at a greater risk of developing T2D compared to individuals with normal weight and low PA levels. One notable limitation of this review was that the conclusions may not apply to individuals with Class II and Class III Obesity due to varying classification cut-offs for obesity and their underrepresentation in the included studies, which often lacked sufficient data for these populations. The current study expands on this by providing a more comprehensive understanding of the relative contributions of PA and BMI to the risk of developing diabetes. The results help enhance the generalisability of the impacts of obesity—paired with various levels of PA—on prevalent diabetes in individuals with Class II and Class III obesity, who were not previously represented in Fogelholm's study. Qin et al. concluded that a positive interactive relationship between PA and obesity exists, meaning that prevention of one factor helps reduce T2D risk not only from that factor but also from the combined effects of both factors [51]. Limitations of these studies [19, 50, 51] include the absence of comprehensive confounding variables, varying measures of PA assessments across studies, and lack of exploration of all categories of obesity. A consistency across the aforementioned studies is that no level of PA was sufficient to mitigate the heightened risk of T2D risk attributable to overweight and obesity [19, 49–54].

While this study has indicated that PA provides a modest protective effect against diabetes compared to BMI, it is also necessary to highlight the indirect relationship between PA and diabetes through its role in lowering BMI. Weight loss achieved from changes to lifestyle behaviors has been recognized as crucial for preventing diabetes, notably in one study with a 16% reduction in diabetes risk for every kilogram of body weight loss [55]. Further research has demonstrated that weight loss achieved from increasing PA and changes to dietary habits provides a significant reduction in the risk of diabetes, even when weight loss is suboptimal [56]. Moreover, PA remains beneficial in T2D prevention and management even if weight loss does not occur, as it helps to maintain a balance between caloric intake and caloric expenditure, thereby preventing further weight gain and subsequent risk of T2D or poor glycemic control. For individuals with T2D, PA also helps to reduce cardiovascular risk by improving blood lipid profiles, lowering blood pressure, and enhancing vascular function, which collectively reduces the likelihood of atherosclerosis and heart disease [57]. Additionally, PA enhances insulin sensitivity, promotes better glycemic control, reduces systemic inflammation, and supports mental well-being by decreasing stress and improving mood, all of which are critical for improving the overall quality of life and well-being [1, 57]. This underscores the importance of promoting regular PA as a cornerstone of T2D prevention strategies, not only for its direct effects on key biomarkers such as insulin resistance, blood pressure, and glucose tolerance but also for its indirect benefits through body fat mass and BMI reduction [58].

### 4.1. Strengths and Limitations

Strengths of this study include the large, nationally representative sample size of US adults, standardised practices of recruitment, and data collection that allow comparison of results with further BRFSS studies and similar explorations. The depth and breadth of the data collected from the BRFSS surveys allowed for the assessment of a range of variables (including sociodemographic and lifestyle factors) and their effect on prevalent diabetes. Furthermore, self-reported cases of diabetes have shown substantial agreement with verified medical records [40]. Another strength of the present study was the presence of a weighting variable that helped correct for nonresponse, stratification, and clustering, improving the generalisability of the findings to the entire US adult population.

While interpreting these results, some limitations need to be considered. Notably, the restrictive cross-sectional nature of this study does not allow for causal exploration between the various exposure variables and the prevalence of diabetes, resulting in temporal ambiguity. While the BRFSS screens for gestational diabetes, it does not differentiate reported diabetes between T1D and T2D. Therefore, it is likely that the true impacts of PA and BMI on T2D reported in the results may have been overestimated or underestimated. The reliance on self-report measures of MVPA is prone to recall bias and may not encapsulate routine PA behaviors [27], with probable overreporting of PA underestimating its true impact on the prevalence of diabetes. This is increasingly likely among individuals who engaged in vigorous PA, as differences between self-reported and objectively measured PA are higher with increasing levels of PA intensity [59]. Furthermore, the protective effect of PA against diabetes in this study was likely to have been confounded by BMI, due to its direct relationship with weight loss and maintenance [60]. Additionally, alcohol consumption was not included as a confounding variable due to the substantial number of respondents with missing data (*n* = 1,199,955). Similarly, recreational drug use was only assessed in the 2017 and 2019 BRFSS surveys, with a large proportion of missing responses, limiting its inclusion in the analysis [21].

While BMI is widely used in population health research due to its easy implementation and cost-effective nature and is associated with morbidity and mortality [61, 62], it has significant limitations as an anthropometric parameter. The absence of other indicators of adiposity, such as waist-to-hip ratio, waist-to-height ratio, body roundness index, and waist circumference, means that central obesity and visceral adiposity—key predictors of T2D [63]—were not assessed in this study. As BMI does not distinguish between fat and lean mass, nor does it account for fat distribution, it may lead to less accurate interpretations of the combined effects of PA and adiposity on the risk of diabetes [64]. This limitation is further compounded by BMI's variability across populations, as thresholds fail to reflect differences in body composition and fat distribution by age, sex, and ethnicity, potentially resulting in misclassification of the prevalence of diabetes, especially among individuals with high muscle mass or those with central adiposity at lower BMI levels [64].

## 5. Conclusion

In summary, the current study adds to the existing foundation of research, indicating that elevated BMI (excess body weight) substantially increases the prevalence of diabetes compared to the modest reductions achievable with PA engagement, particularly among persons affected by Class I, Class II, and Class III obesity. Reducing the burden of overweight and obesity would significantly impact the prevalence and subsequent public health burden of diabetes that currently exists. While only modest benefits were observed from being physically active, the importance of PA as a lifestyle factor should not be overlooked. PA is crucial in reducing diabetes burden not only through its direct relationship with the disease but also through its indirect relationship with reducing excess body weight/BMI and maintenance of a healthy body weight.

Policy approaches to further address the burden of diabetes need to express the importance of a healthy body weight and absence of increased adiposity and supplement current approaches aimed at reducing overweight and obesity. Health professionals treating individuals with overweight or obesity should prioritise healthy weight loss while also emphasizing the importance of being active at any level, supporting the importance of any PA as opposed to being inactive. Future population health studies of the joint effects of PA and BMI on the risk of diabetes utilising objective measures of PA or cardiorespiratory fitness and obesity are needed to verify the findings from this study.

## Figures and Tables

**Table 1:** Weighted characteristics of Behavioral Risk Factor Surveillance System collated sample (2011, 2013, 2015, 2017, and 2019).

**Variable**	**Included sample** ^**b**^	**Weighted** ^**a**^ ** % (95% CI)**
Total	1,913,732	—
*Sociodemographic variables*		
Sex		
Male	843,482	50.6 (50.4–50.7)
Female	1,070,204	49.4 (49.3–49.6)
Age (years)		
18–24	99,375	12.2 (12.1–12.3)
25–34	187,240	16.8 (16.7–17.0)
35–44	230,550	16.6 (16.4–16.7)
45–54	316,699	17.6 (17.5–17.8)
55–64	422,948	16.8 (16.7–16.9)
65+	656,920	20.0 (19.9–20.1)
Race		
White	1,478,074	65.6 (65.4–65.7)
Black	144,249	11.2 (11.1–11.3)
Other	83,784	6.2 (6.1–6.3)
Multiracial	37,042	1.4 (1.4–1.5)
Hispanic	146,178	15.6 (15.5–15.7)
Employment		
Employed	956,142	57.0 (56.9–57.2)
Unemployed	90,966	6.5 (6.4–6.5)
Homemaker	108,261	6.0 (6.0–6.1)
Student	48,054	5.7 (5.6–5.8)
Retired	565,481	18.1 (18.0–18.2)
Unable to work	137,310	6.7 (6.6–6.8)
Education		
Did not graduate high school	141,934	13.1 (13.0–13.3)
Graduated high school	528,172	27.9 (27.8–28.0)
Attended college or technical school	529,082	31.4 (31.2–31.5)
Graduated college or technical school	711,534	27.6 (27.5–27.7)
Marital status		
Married/partnered	1,066,978	56.0 (55.9–56.2)
Widowed/separated/divorced	550,440	20.3 (20.2–20.4)
Single	289,979	23.7 (23.6–23.8)
Number of children		
Zero	1,409,552	63.7 (63.5–65.8)
One	203,530	14.9 (14.8–15.1)
Two	178,697	13.0 (12.9–13.1)
Three or more	116,429	8.4 (8.3–8.5)
*Lifestyle variables*		
Physical activity		
Highly active	654,057	31.8 (31.7–31.9)
Active	346,671	19.0 (18.9–19.2)
Inactive	355,035	20.7 (20.6–20.8)
Nonactive	557,969	28.5 (28.3–28.6)
Muscle-strengthening exercise frequency (sessions per week)		
Zero	1,193,828	59.7 (59.5–59.8)
One	140,655	8.1 (8.0–8.2)
Two	153,038	8.7 (8.6–8.8)
Three or more	403,224	23.5 (23.4–23.6)
Smoking status		
Current smoker	290,871	17.3 (17.2–17.4)
Former smoker	557,427	25.0 (24.9–25.2)
Never smoked	1,056,620	57.7 (57.5–57.8)
Fruit and vegetable consumption (daily servings)		
Zero	9,519	0.6 (0.6–0.7)
One	485,657	28.7 (28.6–28.9)
Two	831,216	42.4 (42.3–42.6)
Three or more	537,104	28.2 (28.0–28.3)
Continuous mean (95% CI)	1.88 (1.87–1.88)	
*Health variables*		
Number of poor mental health days (days per month)		
0	1,275,116	63.9 (63.8–64.1)
1–2	157,580	8.8 (8.7–8.9)
3–6	160,250	9.6 (9.5–9.7)
7–14	96,404	6.1 (6.0–6.2)
15–30	195,813	11.5 (11.4–11.6)
Self-rated health		
Excellent	326,556	18.4 (18.3–18.5)
Very good	631,548	32.4 (32.3–32.6)
Good	587,891	31.1 (31.0–31.3)
Fair	259,526	13.4 (13.3–13.5)
Poor	103,185	4.7 (4.6–4.8)
Number of chronic conditions		
Zero	494,469	34.3 (34.2–24.5)
One	461,369	25.5 (25.4–25.7)
Two	386,440	17.7 (17.6–17.8)
Three or more	571,454	22.5 (22.3–22.6)
Diabetes		
Yes	252,198	10.7 (10.6–10.8)
No	1,661,534	89.3 (89.2–89.4)
Body mass index		
Normal weight	625,851	33.8 (33.7–34.0)
Overweight	701,608	36.2 (36.0–36.3)
Class I obesity	357,043	18.2 (18.0–18.3)
Class II obesity	138,931	7.2 (7.1–7.2)
Class III obesity	90,299	4.7 (4.6–4.7)

Abbreviation: 95% CI, 95% confidence interval.

^a^Data weighted using stratum weight provided by Centers for Disease Control and Prevention.

^b^Included sample of 1,913,732: differing numbers due to missing responses; missing cases as follows: sex = 46 (0.0%), race = 24,405 (1.3%), employment = 7518 (0.4%), education = 3010 (0.2%), marital status = 6335 (0.3%), number of children = 5524 (0.3%), smoking status = 8814 (0.5%), fruit and vegetable consumption = 50,236 (2.6%), self‐rated health = 5026 (0.3%), poorer mental health days = 28,569 (1.5%), and muscle‐strengthening activity frequency = 22,987 (1.2%).

**Table 2:** Adjusted prevalence ratios (APRs)^a,b,c^ for prevalent diabetes for PA, stratified by BMI category.

**BMI** ^**e**^	**Physical activity** ^**d**^				**p** ** value**
	**Highly active**	**Active**	**Inactive**	**Nonactive**	
Normal weight	1	1.05 (1.04–1.05)	1.05 (1.05–1.05)	1.03 (1.03–1.03)	< 0.01
Overweight	1	1.01 (1.01–1.01)	1.06 (1.06–1.06)	1.05 (1.04–1.05)	< 0.01
Class I obesity	1	1.05 (1.05–1.05)	1.03 (1.03–1.03)	1.02 (1.02–1.02)	< 0.01
Class II obesity	1	1.09 (1.09–1.09)	1.04 (1.04–1.04)	1.01 (1.01–1.01)	< 0.01
Class III obesity	1	1.03 (1.03–1.04)	1.05 (1.05–1.05)	1.02 (1.02–1.02)	< 0.01

*Note* : Final sample = 1,913,732.

^a^Data weighted using stratum weight provided by the Centers for Disease Control and Prevention.

^b^Adjusted for age, sex, race, education, employment status, marital status, number of children, smoking status, fruit and vegetable intake, self-rated health, days of poorer mental health, number of additional chronic health conditions, and muscle-strengthening exercise frequency.

^c^95% confidence intervals provided in brackets.

^d^Physical activity categorised as highly active (≥ 300 min/wk), active (150–299.99 min/wk), inactive (1–149.99 min/wk), and nonactive (0 min/wk).

^e^Body mass index categorised as normal weight (18.5–24.99 kg/m^2^), overweight (25–29.99 kg/m^2^), Class I obesity (30–34.99 kg/m^2^), Class II obesity (35–39.99 kg/m^2^), and Class III obesity (≥ 40 kg/m^2^).

**Table 3:** Adjusted prevalence ratios (APRs)^a,b,c^ for prevalent diabetes for BMI, stratified by PA category.

**Physical activity** ^**e**^	**Body mass index** ^**d**^					**p** ** value**
	**Normal weight**	**Overweight**	**Class I obesity**	**Class II obesity**	**Class III obesity**	
Highly active	1	1.54 (1.54–1.55)	2.18 (2.18–2.19)	2.75 (2.75–2.76)	3.32 (3.31–3.32)	< 0.01
Active	1	1.49 (1.49–1.50)	2.16 (2.16–2.17)	2.87 (2.86–2.87)	3.27 (3.26–3.27)	< 0.01
Inactive	1	1.56 (1.56–1.56)	2.10 (2.10–2.10)	2.69 (2.68–2.69)	3.29 (3.28–3.29)	< 0.01
Nonactive	1	1.53 (1.52–1.53)	2.02 (2.02–2.02)	2.48 (2.47–2.48)	2.89 (2.88–2.89)	< 0.01

^a^Data weighted using stratum weight provided by the Centers for Disease Control and Prevention.

^b^Adjusted for age, sex, race, education, employment status, marital status, number of children, smoking status, fruit and vegetable intake, self-rated health, days of poorer mental health, number of additional chronic health conditions, and muscle-strengthening exercise frequency.

^c^95% confidence intervals provided in brackets.

^d^Body mass index categorised as normal weight (18.5–24.99 kg/m^2^), overweight (25–29.99 kg/m^2^), Class I obesity (30–34.99 kg/m^2^), Class II obesity (35–39.99 kg/m^2^), Class III obesity (≥ 40 kg/m^2^).

^e^Physical activity categorised as highly active (≥ 300 min/wk), active (150–299.99 min/wk), inactive (1–149.99 min/wk), and nonactive (0 min/wk).

Final sample =1,913,732.

## Data Availability

The data that support the findings of this study are available in the CDC BRFSS Survey Data and Documentation webpage at https://www.cdc.gov/brfss/data_documentation/index.htm.

## Supporting Information

Supporting Information 1 STROBE checklist: A completed STROBE (Strengthening the Reporting of Observational Studies in Epidemiology) checklist for the submitted manuscript.

Supporting Information 2 Table S1 provides the number of individuals assigned to each combined PA-BMI category for the submitted manuscript, as mentioned in the methods section. Table S2 provides the APRs for the individual effects of PA and BMI on prevalent diabetes, as mentioned in the results section.
