# Supplementary material for: Joint Effects of Physical Activity and Body Mass Index on Prevalent Diabetes in a Nationally Representative Sample of 1.9 Million US Adults
Source: J Diabetes Res. 2025 Mar 8;2025:7466757. doi: 10.1155/jdr/7466757 (PMC11986940; doi:10.1155/jdr/7466757)
Supplement: Supporting Information 1 — STROBE checklist: A completed STROBE (Strengthening the Reporting of Observational Studies in Epidemiology) checklist for the submitted manuscript. [file 7466757.f1.docx]

**Supplementary Document 1. Completed STROBE checklist for cross-sectional studies and analysis plan information**

This checklist was completed using the following formal items recommended for cross-sectional studies from STROBE statement (https://www.strobe-statement.org).

|  | Item No | Recommendation | Completed? | Comments | |
| --- | --- | --- | --- | --- | --- |
| **Title and abstract** | 1 | (*a*) Indicate the study’s design with a commonly used term in the title or the abstract | Yes | | Study design is indicated in methods section of abstract, title uses common terms for measures of adiposity, activity, and study type (prevalent) |
|  |  | (*b*) Provide in the abstract an informative and balanced summary of what was done and what was found | Yes | | Information provided in the study abstract (objective, methods, results and conclusions described) |
| Introduction | | |  | |  |
| Background/rationale | 2 | Explain the scientific background and rationale for the investigation being reported | Yes | | Existing literature explored in background.  Rationale provided at end of background. |
| Objectives | 3 | State specific objectives, including any prespecified hypotheses | Yes | | Statement at the end of the background specifies the objective of the study.  “Therefore, this study aimed to examine the joint effects of PA and BMI on prevalent diabetes in a nationally representative sample of 1.9 million U.S adults while incorporating all classes of obesity and relevant sociodemographic, behavioural and health-related confounding variables.” |
| Methods | | |  | |  |
| Study design | 4 | Present key elements of study design early in the paper | Yes | | Study design is stated in the first paragraph of methods section.  “The Behavioral Risk Factor Surveillance System (BRFSS) is an annual cross-sectional public health survey conducted in the United States that collects data from residents pertaining to health-related risk behaviors, chronic health conditions and the use of preventive health services”. |
| Setting | 5 | Describe the setting, locations, and relevant dates, including periods of recruitment, exposure, follow-up, and data collection | Yes | | Relevant information about the BRFSS survey is provided in the methods section – indicating the location and setting (U.S adult population), which surveys were used (individual years), data collection (PA, BMI and diabetes assessments and categories, explanatory variables). |
| Participants | 6 | (*a*) Give the eligibility criteria, and the sources and methods of selection of participants | Yes | | All individuals who completed the BRFSS survey in 2011, 2013, 2015, 2017 and 2019 were eligible to be included in the study, aside from those who had missing data for physical activity (n=255,692), BMI (n=163,396) or were underweight (n=36,763). |
| Variables | 7 | Clearly define all outcomes, exposures, predictors, potential confounders, and effect modifiers. Give diagnostic criteria, if applicable | Yes | | All outcome and explanatory variables are clearly defined in the methods section. All items have moderate or high levels of reliability and validity. |
| Data sources/ measurement | 8* | For each variable of interest, give sources of data and details of methods of assessment (measurement). Describe comparability of assessment methods if there is more than one group | Yes | | Each survey used the same data collection and measurement methodology for the variables included in the study. |
| Bias | 9 | Describe any efforts to address potential sources of bias | Yes | | The potential for bias was reduced by the weighting of BRFSS respondents using the weighting variable provided by the CDC.  “Each participant was given an individual weighting factor to be used for analyses, effected to help correct for non-response, stratification, and clustering, which increased population representativeness and reduced the risk of bias.” |
| Study size | 10 | Explain how the study size was arrived at | Yes | | The final study size resulted from combining the five BRFSS surveys together and applying the inclusion/exclusion criteria previously identified. |
| Quantitative variables | 11 | Explain how quantitative variables were handled in the analyses. If applicable, describe which groupings were chosen and why | Yes | | All explanatory variables and the chosen categories are detailed in the methods section (*explanatory variables*) |
| Statistical methods | 12 | (*a*) Describe all statistical methods, including those used to control for confounding | Yes | | All statistical methods are described in the *Statistical Analysis* section of the methods. |
|  |  | (*b*) Describe any methods used to examine subgroups and interactions | N/A | | No interaction analysis was necessary. |
|  |  | (*c*) Explain how missing data were addressed | Yes | | Individuals with missing data were not included in the analyses. |
|  |  | (*d*) If applicable, describe analytical methods taking account of sampling strategy | Yes | | The weighting variable provided by the CDC accounted for the BRFSS sampling strategy and helps to correct for non-response, stratification, and clustering of individuals. |
|  |  | (*e*) Describe any sensitivity analyses | N/A | | Not applicable |
| Results | | |  | |  |
| Participants | 13* | (a) Report numbers of individuals at each stage of study—eg numbers potentially eligible, examined for eligibility, confirmed eligible, included in the study, completing follow-up, and analysed | Yes | | This is described at the beginning of the *Results* section, number of individuals included in analysis. Detailed information about missing data for each variable is provided at the bottom of Table 1. Follow-up not applicable due to study design. |
|  |  | (b) Give reasons for non-participation at each stage | N/A | | Not applicable due to study design (cross-sectional) |
|  |  | (c) Consider use of a flow diagram | N/A | | A flow diagram was not deemed as necessary but can be provided if needed |
| Descriptive data | 14* | (a) Give characteristics of study participants (eg demographic, clinical, social) and information on exposures and potential confounders | Yes | | Sample characteristics outlined in first paragraph of results section.  Table 1 provides detailed characteristics for outcome and explanatory variables for the total sample population. |
|  |  | (b) Indicate number of participants with missing data for each variable of interest | Yes | | The number of individuals with missing data for each variable of interest is detailed at the bottom of Table 1. |
| Outcome data | 15* | Report numbers of outcome events or summary measures | Yes | | The crude number and weighted proportion (and 95% confidence intervals) for individuals who reported being diagnosed with diabetes is provided in Table 1. |
| Main results | 16 | (*a*) Give unadjusted estimates and, if applicable, confounder-adjusted estimates and their precision (eg, 95% confidence interval). Make clear which confounders were adjusted for and why they were included | Yes | | All proportions (Table 1) and estimates (Figure 1, Table 2 and 3, Supplementary Table 2) were weighted using the CDC’s weighting variable. Proportions were weighted using the weighting variable, while the estimates (Figure 1 and Supplementary Table 2) were both weighted and adjusted for all variables in the model. |
|  |  | (*b*) Report category boundaries when continuous variables were categorized | Yes | | Category boundaries are displayed in variable headings in the tables, where applicable (age groups, BMI etc.) |
|  |  | (*c*) If relevant, consider translating estimates of relative risk into absolute risk for a meaningful time period | N/A | | Not applicable |
| Other analyses | 17 | Report other analyses done—eg analyses of subgroups and interactions, and sensitivity analyses | N/A | | Not applicable |
| Discussion | | |  | |  |
| Key results | 18 | Summarise key results with reference to study objectives | Yes | | The key findings from this study were described at the beginning of the *Discussion* section, in accordance with the main study aim. |
| Limitations | 19 | Discuss limitations of the study, taking into account sources of potential bias or imprecision. Discuss both direction and magnitude of any potential bias | Yes | | A description of limitations is available under the *Strengths and Limitations* heading of the discussion. |
| Interpretation | 20 | Give a cautious overall interpretation of results considering objectives, limitations, multiplicity of analyses, results from similar studies, and other relevant evidence | Yes | | The results were discussed while considering the study objectives and limitations while also referring to previously conducted studies. |
| Generalisability | 21 | Discuss the generalisability (external validity) of the study results | Yes | | The results of the study were deemed to be generalisable to the adult U.S population (due to the sample size and weighting variable) and discussed appropriately. |
| Other information | | |  | |  |
| Funding | 22 | Give the source of funding and the role of the funders for the present study and, if applicable, for the original study on which the present article is based | Yes | | No relevant funding was obtained for the study and is reported on the title page (as well as the conflict-of-interest disclosure forms). |
| **Additional information on analysis plan** | | |  | |  |
| “For observational studies, authors are required to clearly specify (a) What specific hypotheses the researchers intended to test, and the analytical methods by which they planned to test them; (b) What analyses they actually performed; and (c) When reported analyses differ from those that were planned, authors must provide transparent explanations for differences that affect the reliability of the study's results.” PLoS Med submission guidelines | | | (a) The main study hypothesis was that PA and BMI would have a similar impact on the prevalence of diabetes in the collated BRFSS sample. The analytical methods to test this aim is available in the statistical analysis section of the manuscript.  (b) Adjusted prevalence ratios obtained from Poisson loglinear regression was performed as planned.  (c) The reported analyses did not differ from those that were planned, and the reliability of the study results was not affected. | | |
| “If a prospective analysis plan (from the study's funding proposal, IRB or other ethics committee submission, study protocol, or other planning document written before analyzing the data) was used in designing an observational study, authors must include the relevant prospectively written document with the manuscript submission for access by editors and reviewers and eventual publication alongside the accepted paper. If no prospectively written document exists, authors should explain how and when they determined the analyses being reported.” PLoS Med submission guidelines | | | No formal protocol relative to this study is available.  The chosen analyses were decided at project initiation in accordance with all authors and were revised upon collating the BRFSS surveys into a single dataset. | | |

*Give information separately for exposed and unexposed groups.
