# Supplementary material for: Joint Effects of Physical Activity and Body Mass Index on Prevalent Diabetes in a Nationally Representative Sample of 1.9 Million US Adults
Source: J Diabetes Res. 2025 Mar 8;2025:7466757. doi: 10.1155/jdr/7466757 (PMC11986940; doi:10.1155/jdr/7466757)
Supplement: Supporting Information 2 — Table S1 provides the number of individuals assigned to each combined PA-BMI category for the submitted manuscript, as mentioned in the methods section. Table S2 provides the APRs for the individual effects of PA and BMI on prevalent diabetes, as mentioned in the results section. [file 7466757.f2.docx]

**Supplementary Table 1:** PA^a^-BMI^b^ Categories Table for Behavioral Risk Factor Surveillance System (BRFSS) Sample Population^c^ (2011, 2013, 2015, 2017 and 2019 surveys).

|  |  | **PA Category^a^** | | | |  |
| --- | --- | --- | --- | --- | --- | --- |
|  |  | **Highly Active** | **Active** | **Inactive** | **Nonactive** | |
| **BMI Category^b^** | **Normal Weight** | Highly Active, Normal Weight  (247,369) | Active, Normal Weight  (120,154) | Inactive, Normal Weight  (109,802) | Nonactive, Normal Weight  (148,526) | |
|  | **Overweight** | Highly Active, Overweight  (252,758) | Active, Overweight  (130,814) | Inactive, Overweight  (128,795) | Nonactive, Overweight  (189,241) | |
|  | **Class I Obesity** | Highly Active, Class I Obesity  (104,685) | Active, Class I Obesity  (61,905) | Inactive, Class I Obesity  (69,880) | Nonactive, Class I Obesity  (120,573) | |
|  | **Class II Obesity** | Highly Active, Class II Obesity  (32,966) | Active, Class II Obesity  (21,708) | Inactive, Class II Obesity  (28,347) | Nonactive, Class II Obesity  (55,910) | |
|  | **Class III Obesity** | Highly Active, Class III Obesity  (16,279) | Active, Class III Obesity  (12,090) | Inactive, Class III Obesity  (18,211) | Nonactive, Class III Obesity  (43,719) | |
| ^a^ Physical Activity categorised as Highly Active (≥300 min/wk), Active (150-299.99 min/wk), Inactive (1-149.99 min/wk), Nonactive (0 min/wk).  ^b^ Body Mass Index categorised as Normal Weight (18.5-24.99 kg/m^2^), Overweight (25-29.99 kg/m^2^), Class I Obesity (30-34.99 kg/m^2^), Class II Obesity (35-39.99 kg/m^2^), Class III Obesity (≥40 kg/m^2^).  ^c^ The crude number of individuals is provided in brackets for each of the PA-BMI categories.  Final sample = 1,913,732. | | | | | |  |

**Supplementary Table 2**: Adjusted Prevalence Ratios (APRs) (weighted with and without confounding variables)^a,b^ for Individual Effects of PA^c^ and BMI^d^ on Prevalent Diabetes.

|  | Weighted APR^a^  (95% CI)^e^ | *P* Value for Trend | Weighted APR^a,b^  (95% CI) | *P* Value for Trend |
| --- | --- | --- | --- | --- |
| Physical Activity^c^ | | | | |
| Highly Active | 1.00 (reference) |  | 1.00 (reference) |  |
| Active | 0.93 (0.93-0.93) | <0.001 | 1.06 (1.06-1.06) | <0.01 |
| Inactive | 1.04 (1.04-1.04) | <0.01 | 1.10 (1.10-1.10) | <0.01 |
| Nonactive | 1.79 (1.79-1.79) | <0.001 | 1.11 (1.10-1.11) | <0.05 |
| Body Mass Index^d^ | | | | |
| Normal Weight | 1.00 (reference) |  | 1.00 (reference) |  |
| Overweight | 2.15 (2.14-2.15) | <0.001 | 1.54 (1.54-1.54) | <0.001 |
| Class I Obesity | 3.62 (3.62-3.63) | <0.001 | 2.12 (2.12-2.12) | <0.001 |
| Class II Obesity | 4.95 (4.95-4.96) | <0.001 | 2.66 (2.66-2.66) | <0.001 |
| Class III Obesity | 6.33 (6.32-6.33) | <0.001 | 3.11 (3.11-3.12) | <0.001 |
| ^a^ Data weighted using stratum weight provided by Centers for Disease Control and Prevention.  ^b^ Adjusted for age, sex, race, education, employment status, marital status, number of children, smoking status, fruit and vegetable intake, self-rated health, days of poorer mental health, number of additional chronic health conditions, muscle strengthening exercise frequency.  ^c^ Physical Activity categorised as Highly Active (≥300 min/wk), Active (150-299.99 min/wk), Inactive (1-149.99 min/wk), Nonactive (0 min/wk).  ^d^ Body Mass Index categorised as Normal Weight (18.5-24.99 kg/m^2^), Overweight (25-29.99 kg/m^2^), Class I Obesity (30-34.99 kg/m^2^), Class II Obesity (35-39.99 kg/m^2^), Class III Obesity (≥40 kg/m^2^).  ^e^ 95% CI, 95% Confidence Interval.  Final sample = 1,913,732. | | | | |
